# Asymmetry- and Viscosity-Regulated Atomization of Laminar Impinging Microjets: Morphology Map, Modal Dynamics, and Droplet Statistics

**DOI:** 10.3390/mi17020221

**Published:** 2026-02-07

**Authors:** Xiaoyu Tan, Guohui Cai, Bo Wang, Xiaodong Chen

**Affiliations:** 1 School of Physics, South China Normal University, Guangzhou 511400, China; 2 School of Aerospace Engineering, Beijing Institute of Technology, Beijing 100081, China; 3 School of Mechanical Engineering, Inner Mongolia University of Science and Technology, Baotou 014010, China

**Keywords:** impinging microjets, microscale atomization, liquid sheet breakup, jet-length asymmetry, viscosity effects, regime transition, proper orthogonal decomposition (POD), period doubling, droplet size distribution

## Abstract

Despite decades of studies on symmetric impinging-jet atomization, the combined role of controlled pre-impingement asymmetry and viscosity in setting the instability pathways and droplet statistics of laminar microjets remains insufficiently quantified. The effects of pre-impingement jet-length difference and liquid viscosity on the flow morphologies, instability dynamics, and atomization behavior of laminar impinging microjets are investigated experimentally using high-speed imaging. By systematically varying the jet-length asymmetry and viscosity over a range of Weber numbers, the evolution of liquid-sheet motion and breakup is resolved from synchronized front- and side-view observations. Specifically, the scientific objective of this work is to elucidate how pre-impingement jet-length asymmetry and liquid viscosity jointly regulate the dynamical behavior of laminar impinging microjets, with particular emphasis on regime transitions of liquid-sheet morphologies, the coupling between upper-sheet oscillations and rim instabilities revealed by synchronized multi-view imaging and POD-based frequency analysis and the resulting droplet-size statistics. These aspects address physical questions that have not been systematically resolved in classical impinging-jet studies, which predominantly focus on symmetric configurations or performance-oriented atomization. With increasing Weber number, the flow undergoes a sequence of regime transitions, including merged-jet, liquid-chain, wavy-rim, fishbone, closed-rim, open-rim, and arc-shaped atomization states. The presence and extent of the closed-rim regime depend sensitively on both jet-length asymmetry and liquid viscosity. Increasing jet-length difference accelerates transitions between these regimes, whereas increasing liquid viscosity stabilizes the liquid sheet and shifts the onset of unsteady breakup to higher Weber numbers. Proper orthogonal decomposition is applied to time-resolved image sequences to extract dominant oscillatory modes and their characteristic frequencies. Within the fishbone regime, the oscillation frequency of rim deformation either coincides with that of the upper region of the liquid sheet or appears as its subharmonic, indicating period-doubling behavior under specific combinations of Weber number and jet-length asymmetry. These frequency characteristics govern the spatiotemporal organization of ligament formation and detachment along the sheet rim. In the arc-shaped atomization regime, droplet-size distributions follow a log-normal form, and at sufficiently high Weber numbers, the mean droplet diameter shows only a weak dependence on jet-length asymmetry. These findings provide microscale-regime guidance for tunable droplet formation in open microfluidic jetting and related small-scale multiphase flows. The innovation of this study lies in the systematic use of synchronized multi-view imaging combined with POD-based frequency analysis and droplet statistics to directly connect liquid-sheet oscillations, rim instability dynamics, and breakup organization under controlled geometric asymmetry and viscosity variations. This approach enables a unified physical interpretation of regime transitions and instability mechanisms that cannot be resolved from single-view observations or morphology-based classification alone.

## 1. Introduction

Impinging jets describe a flow configuration in which two liquid jets collide at a prescribed angle, and the outcome of the collision depends on both the jet conditions and operating parameters. Depending on Reynolds number, Weber number, and jet development length, the post-impingement flow can range from stable merged jets or intact liquid structures to thin liquid sheets that exhibit unsteady motion, ligament formation, and eventual atomization into droplets. Owing to the well-defined collision geometry and the flexibility in controlling injection conditions, impinging-jet atomization has been extensively investigated and widely adopted in engineering applications involving liquid-sheet formation and spray generation [1,2,3]. In the microscale regime, laminar impinging microjets provide a controllable platform for liquid-sheet formation and droplet production relevant to small-scale multiphase-flow operations.

Impinging jets are employed in a broad range of practical systems. In aerospace propulsion, impinging-jet atomizers have long served as a core injection strategy in liquid rocket engines, as exemplified by the F-1 engine used in the Saturn V launch vehicle [4,5,6]. Beyond propulsion, impinging jets are utilized in microreactors for nanoparticle synthesis [7,8], inhaler and aerosol generation devices for pulmonary drug delivery [9,10,11,12], and as free liquid-sheet targets for high-repetition-rate laser–-matter interaction experiments [13,14,15,16]. These diverse applications motivate continued experimental and theoretical investigation into the physical mechanisms governing liquid-sheet evolution and atomization.

Similar to other liquid-sheet configurations encountered in practical spray systems, the morphology and stability of sheets formed by impinging jets are governed by the interplay among inertia, surface tension, viscous stresses, and interactions with the surrounding gas phase [17,18,19]. Within this framework, the atomization outcome depends strongly on the flow state of the jets prior to impingement. Whether the incoming jets are laminar or turbulent governs both the initial geometry of the liquid sheet formed at the impingement point and the nature of disturbances introduced into the system [2,3]. For laminar impinging jets, instability development is dominated by interfacial mechanisms intrinsic to the liquid sheet, including rim-dominated capillary instabilities [20,21], interfacial waves associated with Kelvin–Helmholtz-type mechanisms [22], and localized perforations within the sheet interior. With increasing Weber number, these mechanisms may coexist or compete, leading to a variety of breakup pathways characterized by ligament formation, intermittent rupture, and progressive spray development. In contrast, when the jets are turbulent prior to impingement or when the impingement is sufficiently energetic, large-amplitude disturbances generated directly at the collision region, commonly referred to as impact waves, can dominate the breakup process and promote rapid sheet disintegration [23,24,25].

Beyond instability mechanisms, the thickness distribution of liquid sheets formed by impinging jets is a key physical quantity, as it directly influences ligament formation, droplet size, and atomization performance. A number of theoretical models have been proposed to predict sheet thickness based on mass and momentum conservation in the post-impingement radial flow [21,26,27,28]. Classical models, such as the Hasson–Peck formulation, assume an inviscid and uniform radial velocity field and predict a thickness scaling inversely proportional to the radial distance from the impingement point. While these models have been widely used and validated at high Reynolds numbers, they neglect viscous effects within the sheet. Recent experimental and theoretical studies have demonstrated that liquid viscosity can increase sheet thickness and modify its spatial distribution, particularly for microscale and ultra-thin sheets, by inducing momentum exchange and mass transport across adjacent radial regions [16]. These findings indicate that liquid-sheet thickness is not solely determined by impingement geometry but is dynamically regulated by fluid properties.

In addition to thickness distribution, the velocity profile of the jets prior to impingement has been shown to influence the characteristics of the liquid sheet formed by jet collision. Early analytical and theoretical studies often assumed a uniform jet velocity at the nozzle exit, but subsequent work demonstrated that incorporating non-uniform profiles alters predictions of sheet thickness and in-plane velocity distributions [28,29]. In particular, Choo and Kang showed that using experimentally measured, non-uniform velocity profiles leads to thickness and velocity distributions that agree more closely with experimental observations than predictions based on a uniform assumption [28]. Related analyses further demonstrated that parabolic and non-uniform velocity profiles modify the azimuthal variation of sheet velocity and the overall sheet contour [29]. These results highlight that realistic pre-impingement velocity profiles, shaped by nozzle geometry and upstream flow development, play an important role in redistributing momentum within the post-impingement radial flow. Previous studies have shown that asymmetries in the velocity and momentum distribution of impinging jets play a key role in organizing liquid-sheet dynamics and downstream instabilities. Early experiments reported periodic atomization along the sheet rim over a wide range of operating conditions [1,30,31], highlighting the sensitivity of impinging jets to disturbances introduced at or upstream of the collision region. Subsequent work demonstrated that the occurrence of such instabilities depends strongly on liquid viscosity and the flow conditions of the incoming jets [20]. Frontal-view observations further indicated that rim deformations originate near the upper region of the liquid sheet, suggesting that the instability is linked to upstream sheet dynamics rather than being generated locally at the rim. While shear instabilities induced by slight velocity mismatches were proposed as a driving mechanism [21,32], organized instabilities were also observed under nominally symmetric conditions [33]. To reconcile these observations, distinct rim-driven and sheet-driven instability pathways were suggested [34]. Despite these efforts, how velocity asymmetry is introduced, amplified, and transmitted through the liquid sheet, particularly across varying impingement angles and Weber numbers, has remained unclear. Recent work has further shown that instability dynamics in laminar impinging microjets originate from intrinsic oscillations in the upper region of the sheet induced by asymmetric momentum redistribution, with downstream rim breakup acting as a dynamically coupled response rather than an independent instability mechanism [35].

Motivated by these considerations, the present study investigates how upstream flow development and fluid properties jointly regulate the instability dynamics of laminar impinging microjets. Differences in pre-impingement jet length provide a physically transparent and experimentally controllable means of introducing systematic velocity-profile asymmetry without modifying nozzle geometry or nominal inlet conditions. By varying the jet-length difference, the degree of momentum imbalance at impact and the resulting deflection of the upper liquid sheet can be continuously tuned. In addition, liquid viscosity is varied to examine its role in modulating oscillatory dynamics and regime transitions. High-speed front- and side-view imaging is employed to resolve both global sheet motion and local interfacial deformation, while proper orthogonal decomposition is applied to extract dominant oscillatory modes and their characteristic frequencies. Through the combined analysis of flow-regime transitions, modal dynamics, and droplet statistics, this work aims to clarify the dynamical mechanisms by which upstream asymmetry and viscous effects regulate atomization pathways in laminar impinging microjets. This work does not propose a new atomization theory but offers an experimentally grounded clarification of how controllable geometric and rheological factors influence instability evolution and breakup pathways in laminar impinging microjets. By combining synchronized imaging, modal-frequency analysis, and droplet statistics, the study provides a physically interpretable framework that complements classical impinging-jet research, which mainly focuses on symmetric configurations or performance metrics.

## 2. Experiment and Methods

### 2.1. Experimental Setup

**Figure 1:**
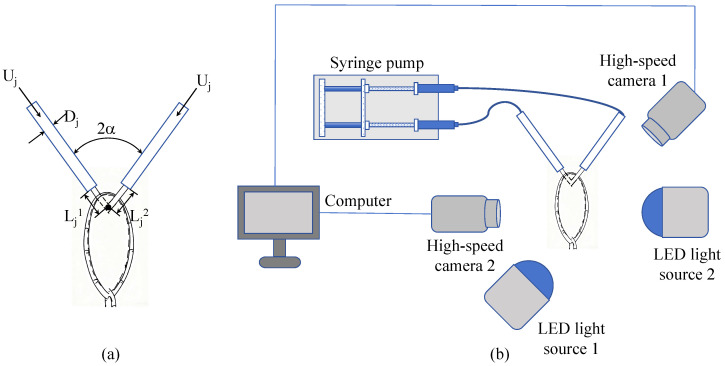
Schematic of the experimental system. (**a**) Impinging-jet geometry showing jet diameter $D_{j}$, jet exit velocity $U_{j}$, impingement angle 2α, and the two pre-impingement jet lengths $\left(L_{j}^{1},L_{j}^{2}\right)$. (**b**) High-speed imaging and LED illumination arrangement.

Figure 1 illustrates the experimental platform used to generate and record the impinging microjets. The system includes a controlled jet-delivery module and a synchronized high-speed optical module, designed to capture both the overall liquid-sheet morphology and the downstream droplet evolution. Figure 1a shows the geometric parameters; two stainless-steel needles are mounted at a fixed impingement angle of $2\alpha =60^{\circ }$, generating two microjets with a nominal diameter $D_{j}$ and exit velocity $U_{j}$. Upstream of the collision point, the two jets have independently adjustable pre-impingement lengths, called $L_{j}^{1}$ and $L_{j}^{2}$. These lengths specify the development distances of the incoming jets and serve as the main geometric parameters controlling asymmetry.

As shown in Figure 1b, a dual-channel precision syringe pump (Pump 11 Pico Plus Elite Dual, Harvard Apparatus, Holliston, MA, USA) delivers liquid through two polyethylene (PE) tubes (outer diameter 4 mm, inner diameter 2 mm) into stainless-steel needles. Front- and side-view imaging is performed using two synchronized high-speed cameras (pco.dimax HS1, PCO Imaging, Kelheim, Germany). Uniform, flicker-free illumination is provided by two high-power LED sources aligned with the optical axes of the cameras. The entire setup is mounted on an optical table to minimize mechanical disturbances and ensure stable, high-fidelity imaging throughout the experiments. For global characterization of the liquid-sheet dynamics, images are recorded at 1000×550 pixels and 9792 fps with a 10 µs exposure time. For downstream droplet-statistics measurements, the frame rate is increased to 13,000 fps while maintaining the same exposure time, ensuring adequate temporal resolution to capture the ligament-to-droplet transition.

The working fluids are deionized water–glycerol mixtures with viscosity $\mu_{l}$ of 2.17 and 4.00 mPa·s. Their densities $\rho_{l}$ are 1060 and 1125 kg/m^3^, respectively. The working fluids contained 0.05% (*w*/*w*) Triton X-100 (Crgent Biotech Co., Ltd., Hangzhou, China) surfactant to control the liquid-air surface tension, which was maintained at $\sigma_{l}=30\;\mathrm{mN}/m$. The jets are issued from stainless-steel nozzles with an inner diameter of $D_{j}$ = 260 µm. The jet exit condition is characterized by the Weber number,

(1) $${\mathrm{We}}_{j}=\frac{\rho_{l}U_{j}^{2}D_{j}}{\sigma_{l}},$$

and the corresponding Reynolds number,

(2) Re=ρlUjDjμl,

where $U_{j}$ is the jet velocity. The relative importance of viscous effects is further quantified by the Ohnesorge number,

(3) $$\mathrm{Oh}=\frac{\mu_{l}}{\sqrt{\rho_{l}\sigma_{l}D_{j}}},$$

which takes values of $\mathrm{Oh}\approx 2.4\times 10^{-2}$ for $\mu_{l}=2.17\;\mathrm{mPa}\cdot s$ and $\mathrm{Oh}\approx 4.3\times 10^{-2}$ for $\mu_{l}=4.00\;\mathrm{mPa}\cdot s$. The experiments cover $14.5\leq {\mathrm{We}}_{j}\leq 700.35$, corresponding to 160≤Re≤1100 for $\mu_{l}=2.17\;\mathrm{mPa}\cdot s$ and 90≤Re≤620 for $\mu_{l}=4.00\;\mathrm{mPa}\cdot s$. Within this Reynolds-number range, the liquid jets remain in a laminar developing state prior to impingement. Accordingly, the following analysis uses ${\mathrm{We}}_{j}$ as the primary parameter representing $U_{j}$.

### 2.2. Proper Orthogonal Decomposition Method

To identify the primary coherent motions contained in the time-resolved images of the impinging microjets, the high-speed recordings are post-processed using the Proper Orthogonal Decomposition (POD) technique [36,37]. Each of the *n* instantaneous frames is reshaped into a vector according to its pixel-intensity distribution I(ξ,t), allowing the temporal evolution of the interface to be represented in the following modal form:

(4) $$I\left(\xi ,t\right)=\sum_{i=1}^{n}a_{i}\left(t\right)\phi_{i}\left(\xi \right).$$

In this formulation, $\phi_{i}\left(\xi \right)$ denotes the spatial basis functions produced by POD, each orthogonal to the others and capturing a distinct energetic pattern of the flow field, while $a_{i}\left(t\right)$ describes the corresponding temporal contribution of each mode.

The decomposition is carried out using a singular-value decomposition (SVD) framework, which is well suited for large image datasets owing to its numerical stability and efficiency. All vectorized snapshots q(ξ,t) are arranged into a data matrix,

(5) $$Y=\{q\left(\xi ,t_{1}\right),q\left(\xi ,t_{2}\right),\ldots ,q\left(\xi ,t_{n}\right)\},$$

and the SVD of *Y* is written as

(6) $$Y=USV^{T}.$$

From this factorization, the spatial POD modes are extracted from the column vectors of *V*, whereas the mode amplitudes are retrieved from

(7) $$a_{i}\left(t\right)=US_{i}.$$

To characterize the dynamical signatures encoded in each mode, the temporal coefficients $a_{i}\left(t\right)$ are further analyzed in the frequency domain using the Fourier transform. The resulting power spectral density (PSD) spectra highlight the dominant oscillation frequencies associated with different coherent structures. Through this procedure, POD serves as an effective means of reducing the dimensionality of the image data while isolating the key spatiotemporal features that dictate the behavior of the liquid sheet.

### 2.3. Measurement of Droplet Sizes

Quantitative droplet-size statistics are extracted from the high-speed shadowgraphy recordings through a threshold-based image-processing pipeline implemented in LaVision DaVis 10.2.1. Before segmentation, each frame is intensity-normalized so that the maximum and minimum grayscale values map to 100% and 0%, respectively. This normalization enhances contrast across the field of view and ensures consistent binarization. A global threshold of 50% is then applied to convert the images into binary form, allowing individual droplet silhouettes to be isolated.

For each detected droplet, the equivalent diameter *d* is calculated based on the measured projected area *A* of the shadow, assuming circular geometry:

$$d=2\sqrt{\frac{A}{\pi }}.$$

To suppress spurious detections introduced by noise or poorly resolved droplets, a minimum object area of five pixels is imposed. This constraint effectively excludes features smaller than the optical resolution. Furthermore, an edge-quality filter is introduced by examining the local intensity gradient near the droplet boundary; objects with boundary slopes below 10% are discarded to eliminate out-of-focus or blurred droplets.

Additional checks confirm that these filtering strategies exert negligible influence on the resulting droplet size probability density functions (PDFs). Lowering the minimum area threshold from five to three pixels leads only to the appearance of a few detections in the smallest diameter range, without altering the location of the distribution peak or its characteristic log-normal shape. These discarded droplets constitute a minor portion of the overall population and are not associated with the primary ligament-mediated breakup events that define the spray dynamics. Consequently, the reported PDFs and their corresponding log-normal fits remain physically representative of the true droplet size distribution.

## 3. Results and Discussion

### 3.1. Effect of $\left(L_{j}^{1},L_{j}^{2}\right)$

**Figure 2:**
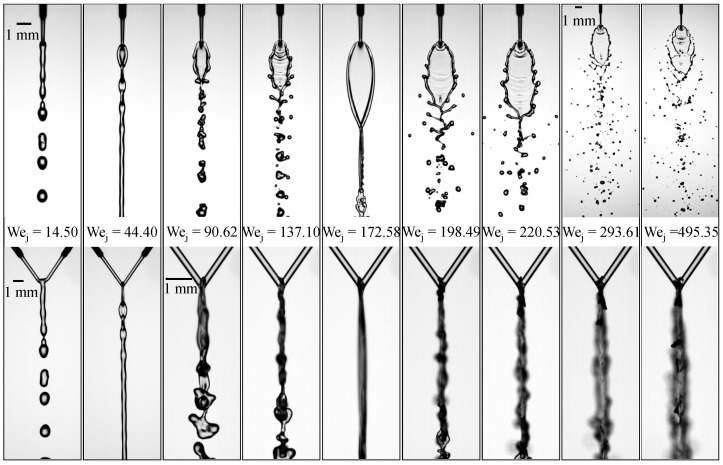
Flow regimes formed by impinging microjets at increasing Weber numbers for the reference case with $\mu_{l}$ of 2.17 mPa·s and nearly symmetric jet lengths $\left(L_{j}^{1},L_{j}^{2}\right)=\left(3.10,3.12\right)\;\mathrm{mm}$.

To examine how upstream geometric differences influence the resulting flow structures, three configurations are constructed based on different combinations of pre-impingement jet lengths $\left(L_{j}^{1},L_{j}^{2}\right)$. Before analyzing the effects of these geometric variations, it is necessary to establish the baseline behavior. Figure 2 presents the flow regimes for the reference case with nearly symmetric jet lengths, $\left(L_{j}^{1},L_{j}^{2}\right)=\left(3.10,3.12\right)\;\mathrm{mm}$, and a liquid viscosity of $\mu_{l}=2.17\;\mathrm{mPa}\cdot s$. The length ratio is defined as $\Lambda =L_{j}^{2}/L_{j}^{1}$, which equals 1.01 for this case. Front- and side-view images are shown to document the flow morphology from both perspectives. As the jet Weber number ${\mathrm{We}}_{j}$ increases, the impinging microjets evolve through a sequence of distinct flow regimes. At low ${\mathrm{We}}_{j}=14.50$, inertia is insufficient to sustain a laterally extended sheet; surface tension promotes coalescence into a thicker downward jet, followed by capillary-driven breakup. The jets merge directly at the impact point, forming a single, thicker downward jet that eventually breaks into droplets of similar size to the incoming jet. This flow pattern is referred to as the *merged-jet* pattern. When ${\mathrm{We}}_{j}$ increases to 44.40, a short and narrow liquid sheet forms, shedding small transverse sub-sheets periodically from its lower edge, resulting in a repeating *liquid-chain* pattern. As ${\mathrm{We}}_{j}$ reaches 90.62, disturbances appear along the sheet rim. Increasing inertia forms a thin sheet, while capillary forces act at the rim. The side-view evidence confirms that rim disturbances originate from oscillations in the upper region of the sheet, indicating a coupling between sheet oscillation and rim instability. Small protrusions emerge from the upper region of the sheet and are carried downstream by convection. The rims along both sides of the sheet converge at the leading edge, followed by complex oscillations that lead to the breakup of the sheet into droplets.

The side-view images confirm that these rim disturbances originate from oscillations in the upper region of the sheet, as observed in previous studies [35]. This flow pattern is referred to as the *wavy-rim* regime [35]. At ${\mathrm{We}}_{j}=137.10$, inertia enhances rim protrusions into finger-like ligaments, while surface tension controls ligament thinning and pinch-off. This is further linked to POD-based frequency analysis, where the dominant rim frequency corresponds to the upper-sheet oscillation frequency, supporting the view that inertial capillary oscillations of the upper sheet drive rim-instability shedding in these regimes. stronger oscillations develop along the upstream rim, and elongated finger-like ligaments appear, signaling the onset of the characteristic *fishbone* regime. In this regime, increasing ${\mathrm{We}}_{j}$ promotes earlier ligament formation and increases the number of ligaments. When ${\mathrm{We}}_{j}$ reaches 172.58, the two jets form a large, smooth, closed liquid sheet with a faint orthogonal sheet beneath it. The upper region of the sheet remains relatively stable but is inclined to the left. This flow pattern is called the *closed-rim* regime. A further increase to ${\mathrm{We}}_{j}=198.49$ introduces oscillations along the rim, giving rise to a fishbone-like pattern again. To distinguish between the two fishbone patterns, the first is referred to as the *first fishbone*, and the second occurrence as the *second fishbone*.

**Figure 3:**
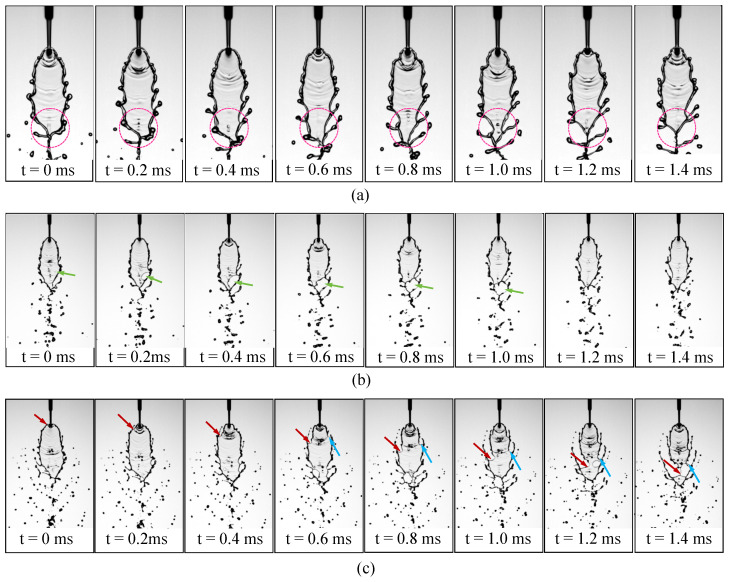
Time-resolved ligament dynamics at three Weber numbers: (**a**) second fishbone pattern at ${\mathrm{We}}_{j}=220.53$; (**b**) open-rim pattern at ${\mathrm{We}}_{j}=293.61$; (**c**) rim-driven rupture and arc-shaped ligament formation at ${\mathrm{We}}_{j}=495.35$.

At ${\mathrm{We}}_{j}=220.53$, the rim instability becomes more pronounced, leading to vigorous ligament formation. Figure 3a presents a series of snapshots showing this process, where oscillations generated near the impingement point develop into finger-like ligaments that propagate along the sheet rim. As indicated by the dashed circle, the lower rim of the liquid sheet remains closed, which is characteristic of the second fishbone morphology.

As ${\mathrm{We}}_{j}$ increases to 293.61, the field of view is shifted downstream to capture the far-downstream droplet region, where the breakup becomes increasingly dispersed and is dominated by ligament fragmentation, as shown in Figure 2. The temporal evolution in Figure 3b shows that at t=0ms, a small perforation appears on the liquid sheet. By t=0.2ms, the perforation grows and reaches the rim, producing ligaments attached to the sheet edge. The opening continues to expand by t=0.4ms, and by t=0.6ms it reaches the rim on the left side, causing the sheet tip to open. At t=0.8ms, the ligaments begin to thin, and they undergo capillary breakup by t=1.0ms. At t=1.2ms, the tip of the sheet starts to close again. This cyclic perforation–reclosure process is clearly distinct from the second fishbone mode, in which the lower boundary of the sheet remains intact throughout. Following the terminology of Bush and Hasha [20], this flow pattern is referred to as an *open-rim* mode, although the sheet tip intermittently alternates between open and closed states.

Finally, at ${\mathrm{We}}_{j}=495.35$, sheet deformation is intensified by hydrodynamic-wave forcing and edge tearing, and atomization results from coupled mechanisms, including wave-induced deformation and ligament growth. Strong oscillations dominate the upper rim, and finger-like ligaments rapidly develop downstream along the sheet, as shown in Figure 2. The temporal evolution in Figure 3c reveals a markedly more violent breakup process: intense oscillations in the upper region of the sheet induce pronounced deformation from t=0ms to t=0.6ms, during which an opening forms near the impingement point and ligament structures emerge, as indicated by the red arrows. The perturbations propagate along the rim and progressively tear the sheet toward its center. As highlighted by the blue arrows, a second opening and associated ligament appear on the opposite side at t=0.6ms, and the two openings subsequently expand toward the centerline of the sheet. By t=1.2ms, a perforation develops below the blue-arrowed opening, further weakening the structure. The openings collapse at t=1.4ms, rupturing the liquid sheet, while the ligaments merge into an arc-shaped structure that eventually disintegrates into droplets downstream. This flow pattern is referred to as the *arc-shaped atomization* regime. We note that, based on our observations under other conditions with similar ${\mathrm{We}}_{j}$, the presence of a perforation is not required for the arc-shaped atomization pattern to occur. This flow pattern closely resembles the upper-sheet-perforation-induced waves recently identified in our study [38], as it is likewise triggered by perforation occurring in the upper region of the liquid sheet.

**Figure 4:**
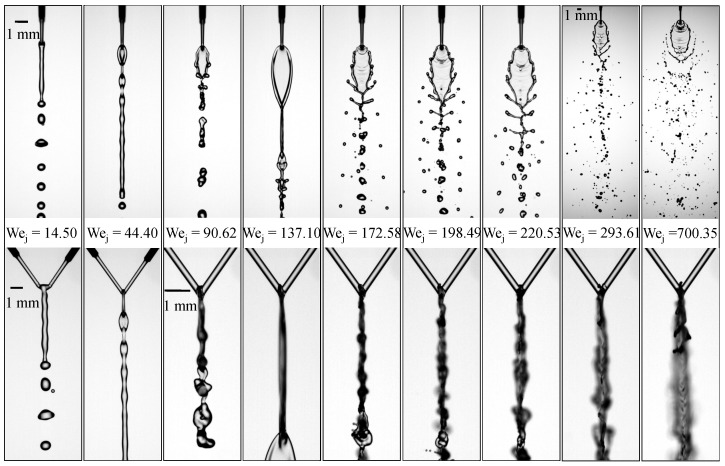
Flow regimes formed by impinging microjets at various Weber numbers for the case at $\mu_{l}$ of 2.17 mPa·s and jet length $\left(L_{j}^{1},L_{j}^{2}\right)=\left(3.10,3.60\right)\;\mathrm{mm}$.

Figure 4 summarizes the evolution of flow morphologies across the full range of tested Weber numbers for the jet–length configuration $\left(L_{j}^{1},L_{j}^{2}\right)=\left(3.10,3.60\right)\;\mathrm{mm}$, for which Λ is 1.16. At very low ${\mathrm{We}}_{j}=14.50$, the jets remain in a merged-jet state and produce axisymmetric droplets through Rayleigh Plateau breakup. When ${\mathrm{We}}_{j}$ increases to 44.40, the column enters a varicose oscillatory dripping regime, forming a repeating liquid-chain pattern with regularly spaced droplets. At ${\mathrm{We}}_{j}=90.62$, pronounced axisymmetric oscillations arise, marking the wavy-rim regime. As ${\mathrm{We}}_{j}$ reaches 137.10, a smooth and fully closed liquid sheet forms immediately after impingement; this closed-rim morphology appears significantly earlier than in the (3.10,3.12)mm case, indicating that stronger upstream asymmetry shortens the wavy-rim regime and accelerates the transition to a stabilized sheet. When ${\mathrm{We}}_{j}$ increases to 172.58, the sheet develops the characteristic fishbone pattern with rim-attached ligaments. At ${\mathrm{We}}_{j}=198.49$, both configurations enter a more vigorous fishbone regime, but their downstream droplet organizations differ markedly: in the (3.10,3.60)mm case, two well-defined and nearly symmetric droplet chains form beneath the ruptured sheet, generated by ligaments detaching at both the tips and roots of the rim; these chains remain close to the centerline at lower Weber numbers and maintain an orderly shedding pattern. In contrast, droplets in the (3.10,3.12)mm case disperse more widely laterally, reflecting stronger upper-rim oscillations that impart larger transverse momentum before ligament breakup, indicating a more intense fishbone regime. As ${\mathrm{We}}_{j}$ increases to 220.53, rim destabilization strengthens, and ligament stretching becomes more pronounced, producing multiple generations of droplets. At ${\mathrm{We}}_{j}=293.61$, the flow transitions into an open-rim breakup regime characterized by periodic sheet ruptures and extensive droplet production through rim erosion and ligament shedding. Finally, at the highest Weber number tested (${\mathrm{We}}_{j}=700.35$), the sheet can no longer maintain coherence and disintegrates into a dense spray field, exhibiting a fully developed arc-shaped atomization structure.

**Figure 5:**
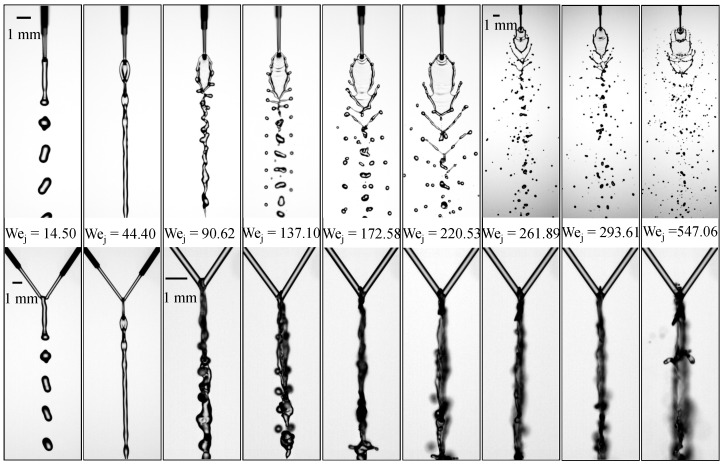
Flow regimes formed by impinging microjetsjets at various Weber numbers for the case at $\mu_{l}$ of 2.17 mPa·s and jet length $\left(L_{j}^{1},L_{j}^{2}\right)=\left(3.10,4.10\right)\;\mathrm{mm}$.

In the $\left(L_{j}^{1},L_{j}^{2}\right)=\left(3.10,4.10\right)$ mm configuration, for which Λ is 1.32, it shows in Figure 5 that a smooth and stable closed liquid sheet never forms. Instead, the flow exhibits only a narrow transitional window in which closed-sheet and fishbone features appear intermittently. This behavior indicates that increasing the jet-length asymmetry amplifies the overall hydrodynamic imbalance, shifting the upper sheet further from the geometric center and strengthening its interaction with the incoming jets, thereby suppressing the establishment of a sustained closed-rim state. At very low ${\mathrm{We}}_{j}=14.50$, the jet remains axisymmetric and undergoes Rayleigh Plateau breakup, producing regularly spaced droplets. When ${\mathrm{We}}_{j}$ increases to 44.40, the system transitions into the liquid-chain pattern. At ${\mathrm{We}}_{j}=90.62$, undulations and perturbations emerge along the rim, marking the onset of the wavy-rim regime. As ${\mathrm{We}}_{j}$ rises to 137.10, these protrusions intensify, and a transitional sheet–ligament structure appears, signaling the emergence of a fishbone-like morphology. By ${\mathrm{We}}_{j}=172.58$, a fully developed fishbone pattern forms, characterized by symmetric rim-attached ligaments that shed droplets on both sides of the sheet.

**Figure 6:**
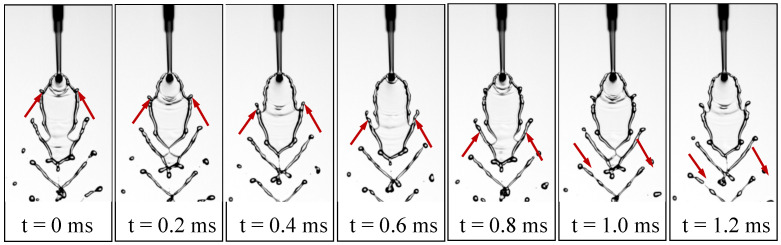
Sequential evolution of the lower-sheet dynamics under ${\mathrm{We}}_{j}$ of 220.53 in the fishbone regime.

Further increases in ${\mathrm{We}}_{j}$ to 220.53 enhance rim destabilization, producing longer ligaments and multisized droplets downstream. Figure 6 illustrates the temporal evolution of the lower-sheet dynamics. At t=0ms, the sheet exhibits a pair of symmetric rim-attached ligaments that begin to thicken near their roots (red arrows). As time progresses to t=0.2–0.6ms, these ligaments elongate steadily while being convected downward, and their bases pull the lower rim outward, producing a V-shaped opening that widens with time. Between t=0.8 and 1.0ms, the ligament tips accelerate downstream, and the tearing fronts on both sides migrate further toward the centerline. By t=1.2ms, the two tearing fronts nearly converge, indicating that the lower sheet edge has been substantially weakened and is approaching rupture. Throughout the sequence, the symmetric outward deformation (highlighted by the arrows) demonstrates how ligament growth directly drives the progressive tearing of the lower sheet, ultimately setting the stage for subsequent open-rim breakup.

As shown in Figure 5, at ${\mathrm{We}}_{j}=261.89$, the lower sheet edge periodically ruptures, causing the flow to transition into an open-rim breakup regime. When ${\mathrm{We}}_{j}$ reaches 293.61, ligament stretching and sheet tearing become more vigorous, generating a dense population of fine droplets. At the highest Weber number examined, ${\mathrm{We}}_{j}=547.06$, the liquid sheet can no longer maintain coherence; both the rim and the sheet interior disintegrate rapidly, resulting in a fully atomized spray cloud composed of fine droplets and fragmented ligament remnants. This condition corresponds to the arc-shaped atomization regime.

**Figure 7:**
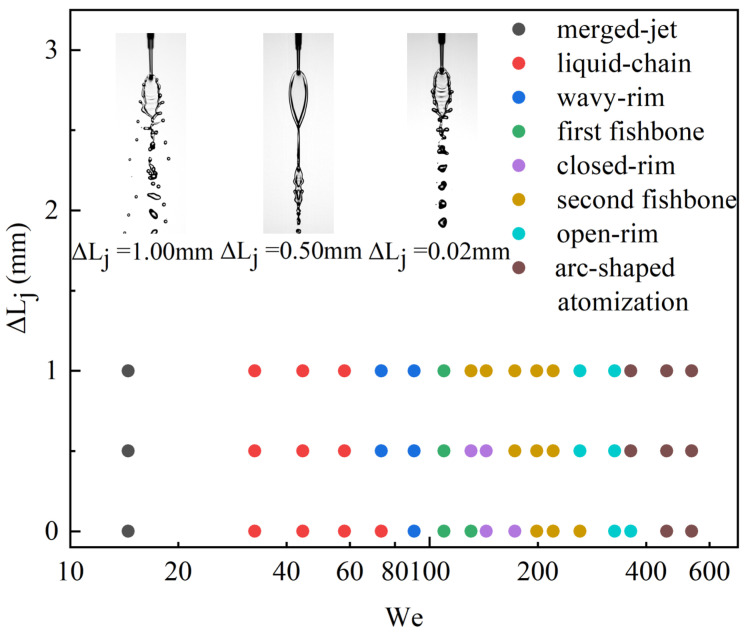
Regime map of liquid-sheet morphologies as a function of the jet Weber number ${\mathrm{We}}_{j}$ for three jet-length differences $\Delta L_{j}=L_{j}^{2}-L_{j}^{1}$ at $\mu_{l}$ of 2.17 mPa·s. Representative liquid-sheet morphologies at ${\mathrm{We}}_{j}=137.10$ are shown for each $\Delta L_{j}$ configuration.

As shown in the regime map in Figure 7, the evolution of liquid-sheet morphologies with increasing Weber number is summarized for the three jet-length differences $\Delta L_{j}=L_{j}^{2}-L_{j}^{1}$ at $\mu_{l}$ of 2.17 mPa·s. Across all tested conditions, the impinging microjets exhibit a well-organized sequence of experimentally observed flow states: merged-jet and liquid-chain structures, first fishbone and closed-rim patterns, second fishbone and open-rim regimes, and ultimately arc-shaped atomization. Jet-length asymmetry introduces a clear destabilizing mechanism: a finite $\Delta L_{j}$ produces unequal pre-impingement momentum fluxes, which laterally displace the upper region of the liquid sheet. As $\Delta L_{j}$ increases, this lateral displacement becomes more pronounced, causing the transitions between successive flow patterns to occur earlier, with the corresponding regime boundaries shifting to lower values of ${\mathrm{We}}_{j}$. The representative snapshots in Figure 7 at ${\mathrm{We}}_{j}=137.10$ further support this interpretation, showing that increasing $\Delta L_{j}$ leads to distinctly different flow patterns, ranging from the second fishbone to the closed-rim and first fishbone structures, as jet-length asymmetry becomes stronger.

### 3.2. Effect of $\mu_{l}$

**Figure 8:**
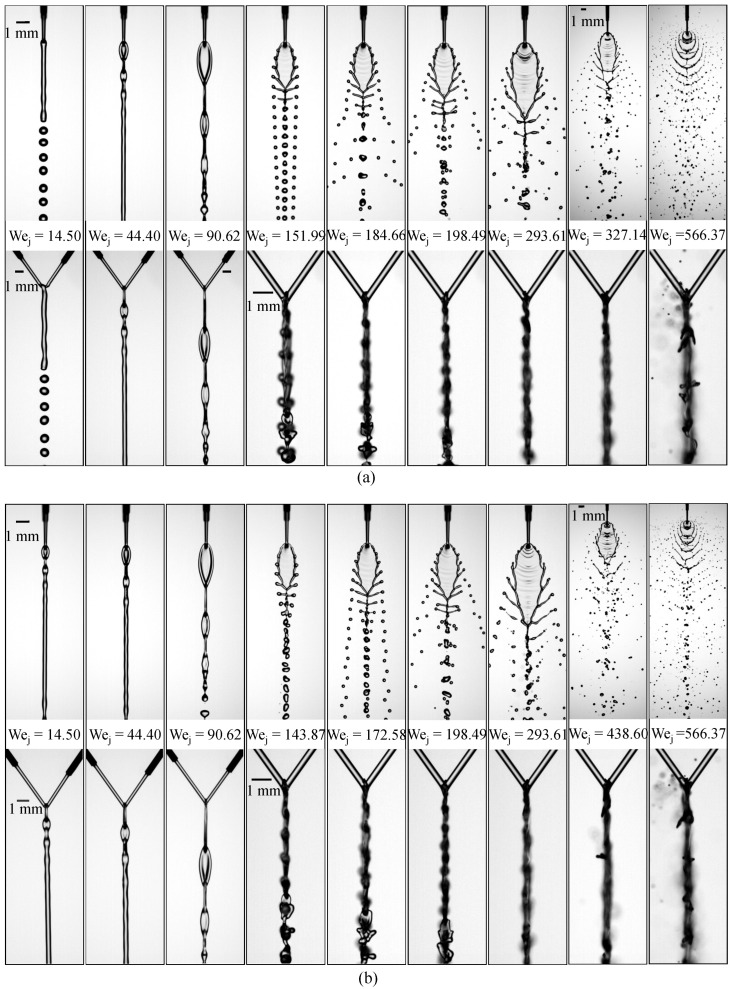
Flow-regime transitions of impinging microjets with liquid $\mu_{l}$ of 4.00 mPa·s over a range of Weber numbers for two jet-length configurations: (**a**) $\left(L_{j}^{1},L_{j}^{2}\right)=\left(3.10,3.60\right)\;\mathrm{mm}$ and (**b**) $\left(L_{j}^{1},L_{j}^{2}\right)=\left(3.10,4.10\right)\;\mathrm{mm}$.

With the influence of jet-length asymmetry clarified in the previous section, we now examine how increasing the liquid $\mu_{l}$ to 4.00 mPa·s alters the evolution of flow morphologies across the full Weber-number range. Figure 8 presents the regime maps for the two asymmetric configurations, $\left(L_{j}^{1},L_{j}^{2}\right)=\left(3.10,3.60\right)\;\mathrm{mm}$ and (3.10,4.10)mm. Overall, the sequence of flow states resembles that observed at 2.17 mPa·s, except that the closed-rim regime is absent, and the transitions between successive patterns occur at noticeably higher ${\mathrm{We}}_{j}$ owing to the enhanced viscous stabilization. For the configuration $\left(L_{j}^{1},L_{j}^{2}\right)=\left(3.10,3.60\right)\;\mathrm{mm}$ shown in Figure 8a, the jets form a merged, axisymmetric column at ${\mathrm{We}}_{j}=14.50$ and undergo Rayleigh–Plateau breakup. When ${\mathrm{We}}_{j}$ increases to 44.40, the breakup enters a varicose oscillatory dripping mode, generating a periodic liquid-chain structure that persists at ${\mathrm{We}}_{j}=90.62$. As ${\mathrm{We}}_{j}$ reaches 151.99, the corrugated fishbone sheet emerges with rim-attached ligaments, and a more vigorous variant of the fishbone regime develops at ${\mathrm{We}}_{j}=184.66$. Further increase to ${\mathrm{We}}_{j}=293.61$ intensifies rim destabilization and promotes ligament stretching, producing multiple droplet generations. At ${\mathrm{We}}_{j}=327.14$, the flow transitions to an open-rim breakup mode characterized by periodic sheet ruptures and rim erosion. At the highest Weber number tested, ${\mathrm{We}}_{j}=566.37$, the sheet loses coherence entirely and disintegrates into a dense spray cloud, forming a fully developed arc-shaped atomization structure. Figure 8b indicates that the overall flow morphology for the $\left(L_{j}^{1},L_{j}^{2}\right)=\left(3.10,4.10\right)\;\mathrm{mm}$ configuration closely follows the sequence observed in Figure 8a for (3.10,3.60)mm. A notable distinction arises at ${\mathrm{We}}_{j}=293.61$, where the downstream finger-like ligaments become significantly more extended when $L_{j}^{2}$ is larger, reinforcing the earlier conclusion that increased geometric asymmetry enhances rim stretching and promotes the development of longer ligaments.

### 3.3. Frequency Characteristics

**Figure 9:**
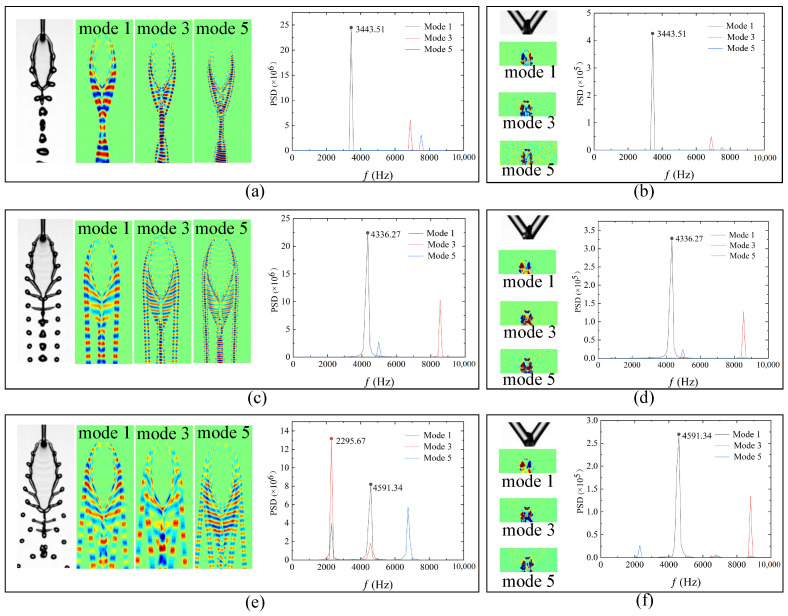
Front- and side-view images of the liquid flow regimes at three Weber numbers for the case with $\mu_{l}$ of 4.00 mPa·s and jet-length configuration $\left(L_{j}^{1},L_{j}^{2}\right)=\left(3.10,3.60\right)\;\mathrm{mm}$. Subfigures correspond to (**a**,**b**) ${\mathrm{We}}_{j}=109.65$, (**c**,**d**) ${\mathrm{We}}_{j}=143.87$, and (**e**,**f**) ${\mathrm{We}}_{j}=157.90$. The associated POD modes and their corresponding frequency spectra are also shown.

Following our previous study [35], POD analysis is conducted to extract the dominant frequency components associated with the fishbone pattern. POD decomposition is applied to both the front- and side-view image sequences using the same number of frames. In our dataset, the POD modes exhibit a typical paired structure: Mode 1 and Mode 2, Mode 3 and Mode 4, Mode 5 and Mode 6, and so on. Within each pair, the spatial structures are highly similar and usually represent the same dominant oscillatory process, with the primary difference being a phase shift rather than a distinct physical mechanism. To avoid redundant presentation of essentially identical dynamical processes while maintaining conciseness and physical interpretability, the first, third, and fifth modes are selected for frequency evaluation. For the side-view data, the spatial domain is restricted to the vicinity of the impingement point to capture the characteristic oscillations of the upper liquid sheet. Figure 9 presents representative results for the case with $\mu_{l}$ of 4.00 mPa·s and jet-length configuration $\left(L_{j}^{1},L_{j}^{2}\right)=\left(3.10,3.60\right)\;\mathrm{mm}$. At ${\mathrm{We}}_{j}=109.65$ (Figure 9a,b), the flow exhibits a wavy-rim behavior. Mode 1 displays a distinct peak at 3443.51 Hz, with a significantly higher PSD amplitude than Modes 3 and 5. This dominant peak corresponds to the rim-oscillation frequency induced by edge instabilities. The same frequency also appears in the side-view results near the upper liquid sheet, demonstrating that the rim oscillation is synchronized with the oscillatory motion of the upper sheet region. This correspondence further verifies that periodic oscillations in the upper liquid sheet constitute the primary mechanism governing the development of the fishbone instability [35].

At ${\mathrm{We}}_{j}=143.87$ (Figure 9c,d), the flow transitions into the fishbone regime. The dominant oscillation frequency extracted from the front-view data is 4336.27 Hz, which again matches the oscillation frequency observed in the upper-sheet region from the side view. This confirms that the fishbone regime at this Weber number remains single-periodic. However, at ${\mathrm{We}}_{j}=157.90$ (Figure 9e,f), the behavior changes. Mode 3 exhibits a higher PSD amplitude than Mode 1, and its dominant frequency is exactly half of that of Mode 1. Although the Mode 1 frequency still coincides with the dominant side-view frequency, the Mode 3 frequency from the front view is half of the corresponding side-view value, indicating the onset of period-doubling behavior [35].

**Figure 10:**
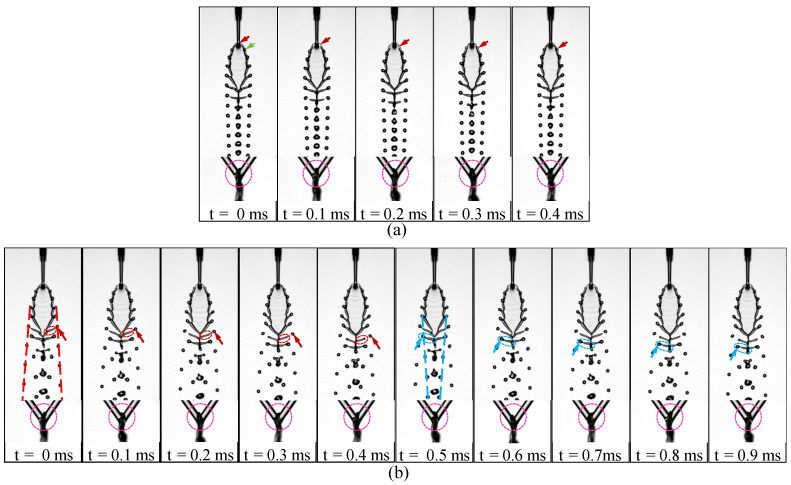
Explanation of the period-doubling phenomenon: (**a**) single-period oscillation, and (**b**) double-period oscillation.

To further clarify the formation mechanism of the fishbone pattern and the onset of its period-doubling bifurcation, the dynamic evolution at the two higher Weber numbers in Figure 9 is examined in Figure 10. At ${\mathrm{We}}_{j}=143.87$ (Figure 10a), the flow exhibits a single-period oscillatory cycle. During one cycle, the upper liquid sheet generates a bead along the rim (red arrows) as time progresses from t=0 to approximately t=0.4ms. At t=0.4ms, the bead indicated by the red arrow occupies nearly the same position as the bead marked by the green arrow at t=0, demonstrating the completion of one full oscillation period. The bead subsequently develops into a ligament that extends downstream and ultimately forms a terminal droplet via pinch-off. As a result of this periodic ligament formation, a queue of droplets, or a droplet string, appears on both the left and right sides downstream. The corresponding side-view dynamics shown beneath Figure 10a also exhibit a complete oscillation cycle, with the red dashed coil highlighting the characteristic left–right swinging motion that reflects the same periodic behavior.

At ${\mathrm{We}}_{j}=157.90$ (Figure 10b), the ligament dynamics become markedly more complex. The temporal evolution reveals that although one full oscillation period elapses in the front view, two droplets are generated during this interval, distinct from the single-droplet formation observed at ${\mathrm{We}}_{j}=143.87$. Correspondingly, two droplet strings develop downstream on both the left and right sides. In Figure 10b, these strings are highlighted using red and blue arrows and guide lines. During the interval from t=0 to 0.4ms, the droplet indicated by the red arrow gradually forms and detaches, with the red ellipse marking the position of the corresponding liquid ligament, and the red dashed lines outlining the resulting droplet string. Between t=0.5 and 0.9ms, a second droplet forms, as shown by the blue arrows and ellipses, producing the second droplet string. This behavior results in two clearly separated droplet strings on both sides of the liquid sheet. The corresponding side-view images beneath Figure 10b show that the upper liquid sheet undergoes two complete oscillations during this interval, confirming the presence of period-doubling. It is further noted that the period-doubling observed in this case differs from that reported in our earlier work [35], as the two distinct droplet strings provide a more direct visualization of the doubled period.

**Figure 11:**
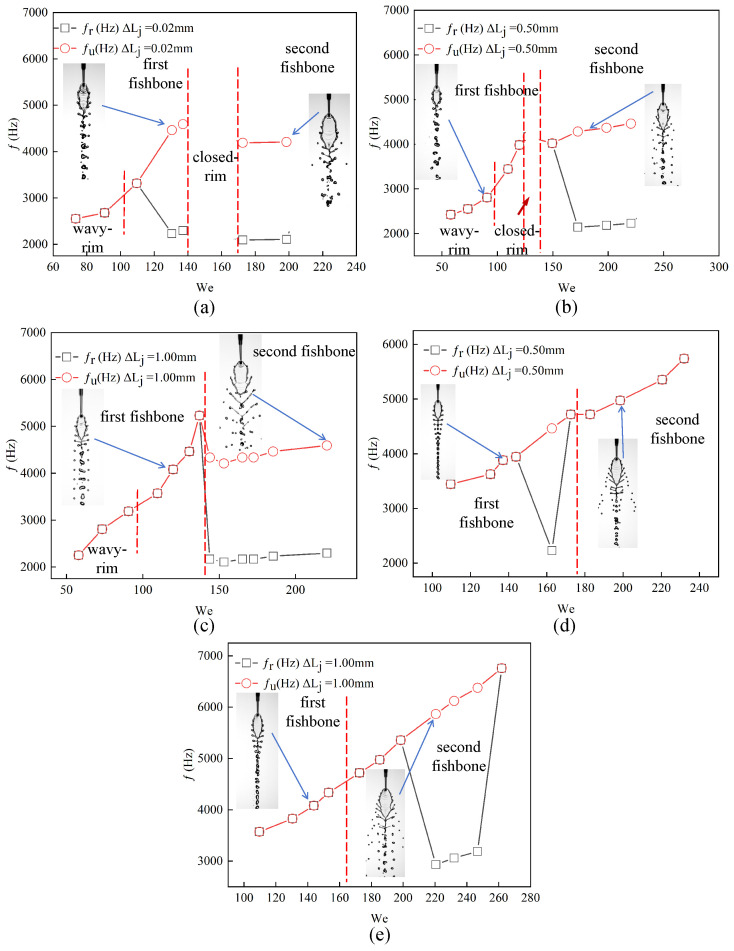
Summary of the frequency relationships between the upper-sheet oscillation frequency $f_{u}$ and the rim frequency $f_{r}$ for different $\mu_{l}$ and $\Delta L_{j}$. Panels (**a**–**c**) show the 2.17 mPa·s cases for $\Delta L_{j}=0.02$, 0.50, and 1.00 mm, respectively, while panels (**d**,**e**) show the 4.00 mPa·s cases for $\Delta L_{j}=0.50$ and 1.00 mm.

Based on these observations, the relationships between $f_{u}$ and $f_{r}$ are further summarized for all operating conditions and both $\mu_{l}$. The dominant frequencies extracted from the POD analysis of the front- and side-view sequences are defined as $f_{r}$ and $f_{u}$, respectively. Figure 11a presents the results for $\Delta L_{j}=0.02\;\mathrm{mm}$ at $\mu_{l}$ of 2.17 mPa·s. At low ${\mathrm{We}}_{j}$, the flow remains in the wavy-rim regime and the frequencies satisfy $f_{r}=f_{u}$. As the Weber number increases, the flow transitions to the first fishbone regime, where single- and double-periodicities appear successively, with $f_{r}=f_{u}$ changing to $f_{r}=f_{u}/2$, demonstrating the presence of period-doubling. When ${\mathrm{We}}_{j}$ approaches approximately 140, the flow enters a closed-rim state in which no frequency. After the flow reaches the second fishbone regime, the relationship remains $f_{r}=f_{u}/2$. Note that $f_{u}$ increases with ${\mathrm{We}}_{j}$ initially and then decreases and then increase slowly when the second fishbone pattern is observed. Figure 11b shows the case with a larger jet-length asymmetry, $\Delta L_{j}=0.50\;\mathrm{mm}$. The ${\mathrm{We}}_{j}$ range in which the closed-rim regime exists becomes significantly narrower, reflecting the destabilizing influence of increasing $\Delta L_{j}$. In this configuration, the first fishbone regime maintains $f_{r}=f_{u}$ with no bifurcation, whereas the second fishbone regime exhibits the transition to $f_{r}=f_{u}/2$. Figure 11c illustrates the condition with an even larger asymmetry, $\Delta L_{j}=1.00\;\mathrm{mm}$. Under this configuration, the closed-rim regime disappears entirely, and the flow transitions only among wavy-rim and fishbone patterns. According to the sudden decrease of $f_{u}$, the boundary between the first and second fishbone patterns can be drawn appropriately. The period-doubling behavior remains confined to the second fishbone regime, where the relationship $f_{r}=f_{u}/2$ holds.

Figure 11d,e present the results for $\mu_{l}$ of 4.00 mPa·s at two different values of $\Delta L_{j}$. Under this condition, the experiments reveal a distinct transition from the fishbone state to the closed-rim state, which allows the critical boundaries between the first and second fishbone regimes to be identified. For the smaller jet-length asymmetry of $\Delta L_{j}=0.50\;\mathrm{mm}$ (Figure 11d), $f_{u}$ increases nearly monotonically over the entire range of ${\mathrm{We}}_{j}$. Both the first and second fishbone regimes exhibit a gradual increase in frequency, while the period-doubling behavior appears only within the second fishbone regime, occurring near ${\mathrm{We}}_{j}\approx 160$. When $\Delta L_{j}$ increases to 1.00 mm (Figure 11e), $f_{u}$ again shows a continuous upward trend with increasing ${\mathrm{We}}_{j}$. Similar to the observations at lower $\mu_{l}$, period-doubling occurs exclusively within the second fishbone regime, with no frequency bifurcation detected in the first fishbone regime.

### 3.4. Droplets Characteristics

The atomization performance of impinging microjets at different impact angles and Weber numbers is evaluated using the mean droplet diameter. A generalized $d_{mn}$ mean, defined through the *m*-th and *n*-th moments of the measured droplet diameters, is expressed as

(8) $$d_{mn}={\left(\frac{\sum_{i=1}^{N}d_{i}^{\;m}}{\sum_{i=1}^{N}d_{i}^{\;n}}\right)}^{\frac{1}{m-n}},$$

where $d_{i}$ denotes the diameter of the *i*-th droplet and *N* is the total number of sampled droplets. When m=1 and n=0, this expression reduces to the arithmetic mean diameter $d_{10}$. When m=3 and n=2, it yields the Sauter mean diameter $d_{32}$, which characterizes the volume-to-surface-area ratio of the spray.

**Figure 12:**
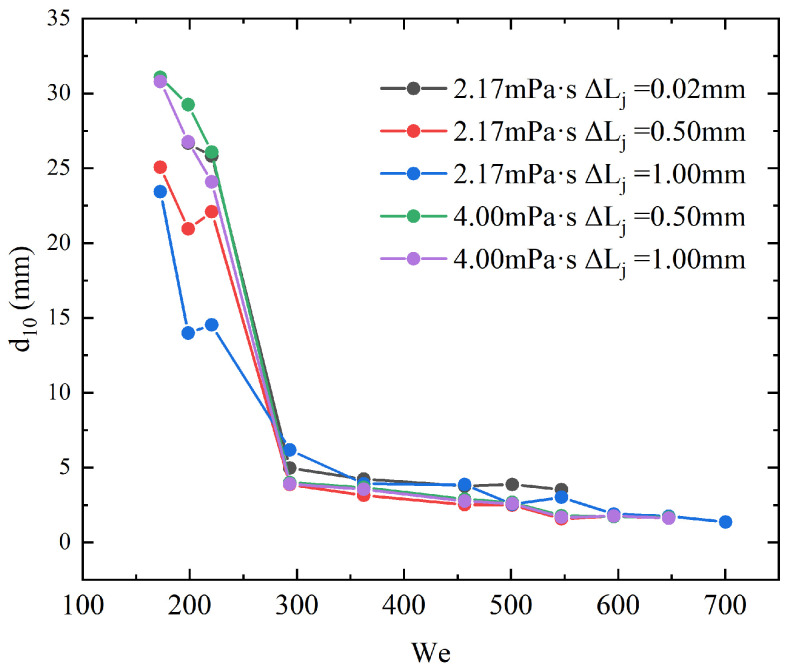
Evolution of the mean droplet diameter $d_{10}$ with the jet Weber number ${\mathrm{We}}_{j}$ for five operating conditions involving two $\mu_{l}$ and three jet-length asymmetries $\Delta L_{j}$.

As shown in Figure 12, the variation of $d_{10}$ with ${\mathrm{We}}_{j}$ is presented for the five operating conditions. A clear inverse trend is observed: increasing ${\mathrm{We}}_{j}$ substantially reduces the mean droplet diameter. This behavior indicates that a higher ${\mathrm{We}}_{j}$ intensifies jet impingement, promotes earlier destabilization of the liquid sheet, and leads to more complete atomization. At relatively low ${\mathrm{We}}_{j}$, the differences among the three $\Delta L_{j}$ configurations at $\mu_{l}$ of 2.17 mPa·s are pronounced. Smaller $\Delta L_{j}$, corresponding to higher geometric symmetry, produces noticeably larger mean droplet diameters because symmetric jets form a smoother and more stable liquid sheet, delaying sheet rupture and yielding larger droplets after breakup. As ${\mathrm{We}}_{j}$ increases, however, the mean droplet diameters for all three configurations rapidly converge and fluctuate only within a narrow range. In this high-${\mathrm{We}}_{j}$ regime, breakup is dominated by upper-sheet-perforation-induced waves, whose influence overwhelms the geometric effects associated with $\Delta L_{j}$. Consequently, the role of jet-length asymmetry in determining droplet size becomes significantly weaker at high ${\mathrm{We}}_{j}$ compared with the low-${\mathrm{We}}_{j}$ regime. For $\mu_{l}$ of 4.00 mPa·s, the differences between the two $\Delta L_{j}$ configurations remain small across the entire ${\mathrm{We}}_{j}$ range, indicating that $\mu_{l}$ further suppresses the influence of jet-length asymmetry on droplet size.

**Figure 13:**
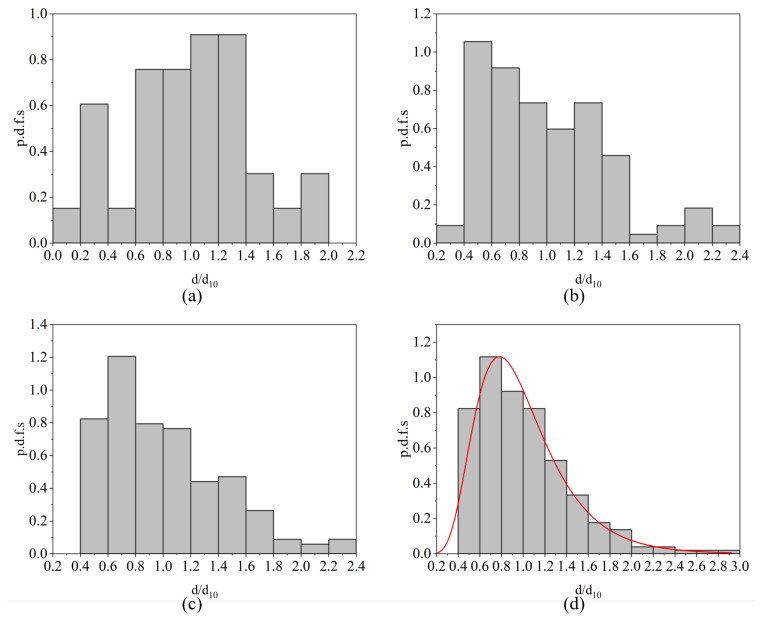
Evolution of the probability density functions of droplet diameters for the case with $\mu_{l}$ of 2.17 mPa·s and a jet-length range $\left(L_{j}^{1},L_{j}^{2}\right)=\left(3.10,4.10\right)\;\mathrm{mm}$. Subfigures correspond to (**a**) ${\mathrm{We}}_{j}=198.49$, (**b**) ${\mathrm{We}}_{j}=362.48$, (**c**) ${\mathrm{We}}_{j}=501.30$, and (**d**) ${\mathrm{We}}_{j}=700.35$.

Figure 13 shows the probability density functions (p.d.f.s) of droplet diameters for the case with $\mu_{l}$ of 2.17 mPa·s and jet lengths in the range $\left(L_{j}^{1},L_{j}^{2}\right)=\left(3.10,4.10\right)\;\mathrm{mm}$ at different Weber numbers. At ${\mathrm{We}}_{j}=198.49$ (Figure 13a), droplet breakup occurs predominantly beneath the liquid sheet, and the resulting size distribution exhibits a complex multimodal structure, with medium-sized droplets accounting for most of the population. When ${\mathrm{We}}_{j}$ increases to 362.48 (Figure 13b), the proportion of medium-sized droplets starts to decrease, while smaller droplets become more abundant, and a small fraction of relatively large droplets begins to appear. At ${\mathrm{We}}_{j}=501.30$ (Figure 13c), the distribution evolves toward a more clearly unimodal shape, with droplet sizes becoming increasingly concentrated around a dominant peak. By ${\mathrm{We}}_{j}=700.35$ (Figure 13d), the distribution is well described by a log-normal function: a fully developed unimodal, right-skewed profile is observed, in which most droplets fall within a narrow size range while a small number of large droplets form a long tail extending to the right. This type of right-skewed, unimodal behavior is characteristic of fully developed atomization and is commonly modeled using the log-normal distribution in spray and atomization studies. The normalized probability density function of the log-normal distribution is expressed as

(9) $$P\;\left(X=\frac{d}{d_{10}},\;\beta ,\;X_{0}\right)=\frac{1}{X\;\beta \sqrt{2\pi }}\exp\;\left[-\frac{{\left(\ln X-\ln X_{0}\right)}^{2}}{2\beta^{2}}\right],$$

where *d* denotes the droplet diameter, $d_{10}$ is the mean droplet diameter, $X=d/d_{10}$ is the normalized droplet size, and β and $X_{0}$ are fitting parameters. In the impact-wave-dominated atomization regime, corresponding to the fully developed breakup at high ${\mathrm{We}}_{j}$, the measured droplet-size distribution exhibits a clear log-normal character that is well captured by this functional form.

**Figure 14:**
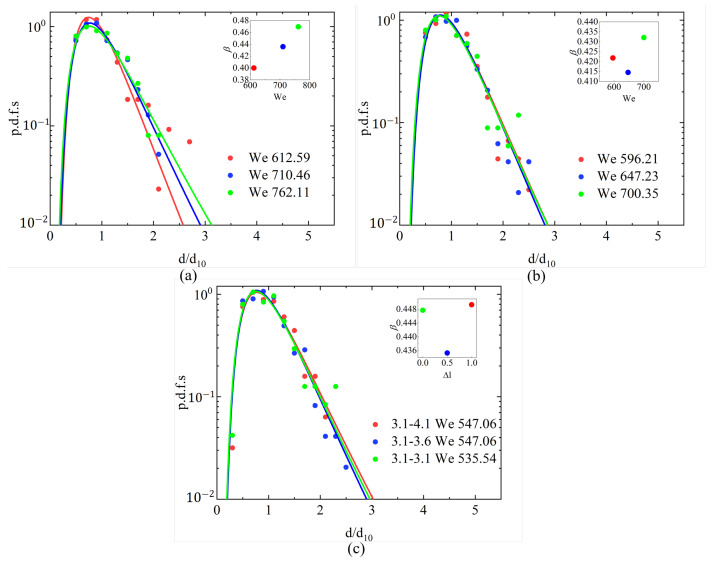
Log-normal p.d.f.s of droplet diameters. (**a**) Droplet-size p.d.f.s for three representative ${\mathrm{We}}_{j}$ at $\mu_{l}$ = 2.17 mPa·s. (**b**) Droplet-size p.d.f.s for three representative ${\mathrm{We}}_{j}$ at $\mu_{l}$ = 4.00 mPa·s. (**c**) Droplet-size p.d.f.s for different $\Delta L_{j}$ at approximately the same ${\mathrm{We}}_{j}$ for $\mu_{l}$ = 2.17 mPa·s.

Figure 14a,b show the fitted log-normal p.d.f.s for three representative Weber numbers under the conditions of 2.17 mPa·s 4.00 mPa·s conditions, respectively. In both cases, the droplet-size distributions collapse onto nearly identical curves, regardless of ${\mathrm{We}}_{j}$. This indicates that once the breakup enters the arc-shaped atomization regime, the influence of the Weber number on the droplet-size statistics becomes negligible. Consistently, the fitted parameter β remains confined within a narrow and physically reasonable interval, further supporting the robustness of the log-normal behavior. Figure 14c compares the droplet-size p.d.f.s for different jet-length differences under 2.17 mPa·s at approximately the same Weber number. A remarkable similarity is observed across all configurations once atomization is fully developed. Although the case with a jet-length difference $\Delta L_{j}=0.02\;\mathrm{mm}$ yields the largest β value, the deviation among all three configurations remains very small. This confirms that, in the fully atomized regime, the jet-length difference has only a negligible effect on the resulting droplet-size distribution.

## 4. Conclusions

This work is motivated by the need for a systematic understanding of how controlled pre-impingement asymmetry and liquid rheology reshape instability pathways in laminar impinging-jet systems beyond the canonical symmetric configurations commonly addressed in classical studies. This study investigated the effects of pre-impingement jet-length asymmetry and liquid viscosity on the instability dynamics and atomization behavior of laminar impinging microjets over a wide range of Weber numbers ${\mathrm{We}}_{j}$. By combining synchronized high-speed front- and side-view imaging, proper orthogonal decomposition, and droplet-size statistics, the roles of upstream momentum imbalance and rheological effects in governing liquid-sheet evolution, oscillatory dynamics, and breakup pathways were clarified. In particular, the study is designed to address how geometric asymmetry and viscosity jointly influence regime transitions, oscillatory coupling mechanisms, and the resulting atomization outcomes.

With increasing ${\mathrm{We}}_{j}$, the impinging microjets exhibit a well-organized sequence of flow regimes, including merged-jet, liquid-chain, wavy-rim, first fishbone, closed-rim, second fishbone, open-rim, and ultimately a fully developed arc-shaped atomization regime. For a fixed liquid viscosity, increasing the jet-length difference $\Delta L_{j}$ systematically accelerates the transitions between successive regimes and shifts the corresponding boundaries toward lower ${\mathrm{We}}_{j}$. This behavior reflects the destabilizing influence of geometric asymmetry, which introduces unequal pre-impingement momentum fluxes and induces a lateral deflection of the upper region of the liquid sheet, thereby enhancing rim stretching, ligament growth, and premature breakup. In contrast, increasing the liquid viscosity stabilizes the sheet, delays regime transitions to higher ${\mathrm{We}}_{j}$, and suppresses the persistence of a stable closed-rim morphology, resulting in a smoother, more gradual evolution of flow patterns.

Proper orthogonal decomposition (POD)-based frequency analysis reveals a strong dynamical coupling between oscillations in the upper region of the liquid sheet and rim deformation in the wavy-rim and fishbone regimes. For all conditions examined, the dominant rim frequency $f_{r}$ either coincides with the upper-sheet oscillation frequency $f_{u}$ or appears at its subharmonic, indicating that rim dynamics are governed by oscillatory motions originating near the impingement region rather than by a purely local rim instability. At low Weber numbers and near the onset of fishbone formation, the response is predominantly single-periodic with $f_{r}=f_{u}$, whereas increasing the Weber number leads to subharmonic responses and period-doubling, characterized by $f_{r}=f_{u}/2$. The emergence and extent of period-doubling depend on both jet-length asymmetry and liquid viscosity. Increasing asymmetry suppresses the closed-rim regime and progressively confines period-doubling to higher-${\mathrm{We}}_{j}$ fishbone states, while higher viscosity lowers the overall oscillation frequency and narrows the range of ${\mathrm{We}}_{j}$ over which periodic behavior is observed, without altering the qualitative frequency organization. Transitions between single- and double-periodic states are accompanied by clear changes in breakup morphology: single-period oscillations produce one droplet string on each side of the sheet, whereas period-doubling leads to two alternating droplet strings, directly linking frequency bifurcation to ligament formation and droplet organization.

In the fully developed atomization regime, droplet-size distributions are well described by a log-normal form. At sufficiently high Weber numbers, the mean droplet diameter becomes nearly insensitive to jet-length asymmetry, indicating that breakup is dominated by upper-sheet-perforation-induced waves that overwhelm geometric effects. In contrast, below the sheet-to-spray transition, geometric symmetry plays a more prominent role, with smaller $\Delta L_{j}$ producing larger mean droplet diameters owing to the formation of smoother and more stable liquid sheets prior to breakup.

Overall, the present results demonstrate that jet-length asymmetry and liquid viscosity exert strong and complementary influences on impinging-jet atomization by regulating upstream sheet oscillations, rim instability dynamics, and breakup pathways. The combined analysis of flow-regime transitions, POD-based frequency characteristics, and droplet statistics provides a unified physical framework for interpreting instability mechanisms and atomization behavior in laminar impinging-jet systems, with direct relevance to the modeling and control of practical spray processes under geometric and rheological variations. Rather than proposing a new atomization theory, this work provides experimentally grounded physical evidence and a quantified morphology–dynamics map that clarifies how controllable geometric and rheological parameters govern instability development and breakup organization in laminar impinging microjets.

## Data Availability

The data that support the findings of this study are available from the corresponding author upon reasonable request.
